# Perspectives on Ease of Use and Value of a Self-Monitoring Application to Support Physical Activity Maintenance among Individuals Living with and beyond Cancer

**DOI:** 10.3390/curroncol31030120

**Published:** 2024-03-19

**Authors:** Manuel Ester, Meghan H. McDonough, Mannat Bansal, Julianna Dreger, Julia T. Daun, Margaret L. McNeely, Thompson Luu, S. Nicole Culos-Reed

**Affiliations:** 1 Faculty of Kinesiology, University of Calgary, Calgary, AB T2N 1N4, Canada; meghan.mcdonough@ucalgary.ca (M.H.M.); mannat.bansal@ucalgary.ca (M.B.); jtdaun@ucalgary.ca (J.T.D.); thompson.luu@ucalgary.ca (T.L.); 2 Department of Physical Therapy, University of Alberta, Edmonton, AB T6G 2R3, Canada; mmcneely@ualberta.ca; 3 Department of Oncology, University of Alberta, Edmonton, AB T6G 2R3, Canada; 4 Rehabilitation Medicine, Cross Cancer Institute, Edmonton, AB T6G 1Z2, Canada; 5 Department of Oncology, Cummings School of Medicine, University of Calgary, Calgary, AB T2N 1N4, Canada; 6 Department of Psychosocial Resources, Tom Baker Cancer Centre, Cancer Care, Alberta Health Services, Calgary, AB T2N 4N2, Canada

**Keywords:** exercise, physical activity, oncology, cancer care, behavior change, electronic health, mobile health, digital technology, qualitative research

## Abstract

Background: Physical activity (PA) can improve the physical and psychosocial health of individuals with cancer, yet PA levels remain low. Technology may address PA maintenance barriers in oncology, though the intervention effectiveness to date remains mixed. Qualitative research can reveal the nuances of using technology-based PA maintenance tools. The present study aimed to understand the perspectives of individuals with cancer on using an app to support PA maintenance. Methods: Individuals were interviewed after using a self-monitoring app for 24 weeks, asking about their app use, ease of use, and perceived value for supporting PA. Analyses were guided by an interpretive description. Results: Eighteen individuals were interviewed. The participants were 37–75 years old; lived in seven Canadian provinces/territories; identified as White, South Asian, or Indigenous; and had eight different cancers. Four themes were developed: some did not need the app to stay physically active, some valued the app for helping them maintain their PA, the user experience ranged from intuitive to confusing, and the time burden of app use ranged from acceptable to overwhelming. Conclusions: The participants provided insights on using a self-monitoring app to improve PA maintenance in oncology. Work is needed to capture additional perspectives and apply findings to the development of technology-based PA maintenance tools.

## 1. Introduction

Physical activity (PA) can improve the physical and psychosocial health of individuals living with and beyond cancer, and has been linked to increased survival and reduced recurrence in some cancers [1,2]. However, few individuals meet cancer-specific exercise guidelines and PA levels commonly decline after the completion of exercise oncology programs, which often last between 8 and 12 weeks [3,4,5,6,7]. These findings indicate that many exercise oncology program participants require ongoing support to maintain PA in the long term [3,8].

Given the challenges of scaling and sustaining PA maintenance interventions and addressing PA barriers, technology-based tools are being explored to support PA maintenance [9,10,11,12]. The key advantages of technology-supported interventions include their suitability for low-cost remote delivery, increasing reach and scale-up potential, and unique features such as enhanced self-monitoring and feedback on behavior, which can support PA maintenance in oncology [13,14,15,16,17]. The use of technology to support PA maintenance is particularly relevant for rural and remote cancer populations, who have greater PA barriers and more limited access to local PA resources compared to those in urban areas [18,19,20]. However, a recent review found that the effectiveness of technology-based interventions to change PA behaviors in this population was mixed, with many interventions reporting no significant effect on PA levels and no interventions specifically targeting PA maintenance among rural and remote individuals [11].

Qualitative investigations may provide an insight into how people understand why and how such interventions do or do not support PA behavior change [12,21,22,23,24,25,26,27,28,29,30]. Past research emphasizes the potential value of technology to support PA, by increasing accountability and providing feedback, and important features such as personalized easy-to-use interfaces and social components [21,22,23,24,25,26,27,28,29,30]. The challenges for these technologies include low data accuracy, time-consuming use, and poor ease of use, especially among individuals with lower technology literacy. However, studies to date have focused primarily on urban individuals in the early stages of PA behavior change (i.e., PA adoption) [23,24,25,26,27,28,29,30], and conducted interviews after limited short-term engagement (i.e., 1–8 weeks of app use) with technology-based interventions [23,24,25,26,27,28,29].

To address these limitations in the work to date, this study was guided by the technology acceptance model, a widely applied model to understand technology use in a variety of contexts [31,32]. The model, which is based on the theory of planned behavior, posits perceived ease of use and perceived usefulness as the two central factors influencing technology use. Each factor is impacted by key determinants including the output quality and relevance for perceived usefulness, and the enjoyment and usability for ease of use. External contexts (e.g., experience, social influence) are included in the model as potential moderators of the perceived usefulness and ease of use.

Based on these noted limitations in prior qualitative research, important knowledge gaps remain related to rural and remote cancer populations, use of the technology beyond the initial PA adoption phase, and collecting feedback after a prolonged engagement with the technology. Therefore, the purpose of the present study was to gather the perspectives of rural and remote individuals living with and beyond cancer on the use of a mobile app to promote PA maintenance. The research questions were as follows: (1) “What are participant perspectives on the ease of use of the self-monitoring app?”, and (2) “What are participant perspectives on the usefulness of the self-monitoring app to support PA maintenance?”.

## 2. Materials and Methods

Participants were recruited from the intervention group of a 2-arm cluster randomized controlled trial (RCT) of an app-based self-monitoring intervention to support PA maintenance (i.e., long-term PA habits up to and beyond 24 weeks), which was embedded within the Exercise for Cancer to Enhance Living well (EXCEL) effectiveness-implementation study [33,34,35]. The RCT was prospectively registered and its protocol was published prior to the study starting (NCT 04790578) [33]. Participants used the self-monitoring app to track their PA and health (i.e., energy, fatigue, symptoms, and other personally relevant factors) for the 24-week study period. Semi-structured 1-to-1 interviews were conducted directly after study completion to discuss participant perspectives on the ease of use and usefulness of the self-monitoring app to support PA maintenance, and to better understand the varied contextual factors that may have impacted these perspectives.

### 2.1. Qualitative Methodology

This qualitative study was guided by interpretive description methodology, a well-established methodology in applied health research based on a constructivist philosophy [36,37]. Constructivist ontology posits that multiple socially constructed realities exist, shaped by contextual factors and lived experiences of each individual. Furthermore, the interaction between researchers and individuals is seen as essential to understanding both common and divergent aspects of these realities, valuing subjectivity and an inductive process when developing knowledge. These aspects of constructivism were applied to guide the study design, interview guide development, interview delivery, and data analysis and reporting. Theoretical scaffolding is a key component of interpretive description. For the present study, past exercise oncology research, the technology acceptance model, and the researcher’s practice-based knowledge were used as the theoretical scaffold.

### 2.2. Participants and Interviews

Interviews were conducted by the first author (ME) directly after each of the 24-week app usage periods starting in April 2021, September 2021, January 2022, and April 2022. Convenience sampling was used for the first round of interviews, to include all those interested. For subsequent interview rounds, purposive sampling was used to collect a range of perspectives considering demographic backgrounds (age, gender, cancer diagnosis, treatment type, self-reported PA, baseline technology use) and experiences with the self-monitoring app (mobile app usability questionnaire ratings, app usage over time). The semi-structured interview guide was informed by previous exercise oncology studies and the technology acceptance model, and was developed by the first author (ME) with the help of experienced qualitative and mixed methods researchers (NCR, MHM), featuring open-ended questions about the self-monitoring app (ease of use, perceived usefulness) and PA maintenance behaviors [23,24,25,26,27,28,29,30,31,32]. The interview guide was piloted and iteratively revised by the research team prior to participant interviews. A copy of the interview guide is available upon request. Interviews lasted between 30 and 65 min.

Discussions between authors (ME, NCR, and MHM) were held to guide purposive sampling decisions as well as to adjust the interview guide to ensure that additional unique insights were collected. For example, after 2 rounds of interviews, the interview guide was adapted to increase emphasis on PA maintenance behaviors, while sampling for round 3 focused on recruiting outside of female breast cancer participants and increasing geographic representation. Interviews were conducted at a time convenient for participants via ZOOM videoconferencing and audio was recorded for analysis.

### 2.3. Data Analysis

Audio recordings were transcribed verbatim by two authors (ME and TL) and imported into NVivo 12 for analysis [38]. A brief summary was written for each transcript, describing overall impressions and key concepts shared by participants. The first author (ME) coded each transcript to identify key concepts relevant to the research question. Codes were refined through iterative rounds of reviewing the codes, individual transcripts, theoretical scaffolding, and discussions with co-authors. Themes were developed inductively from the codes by the first author (ME), in collaboration with the senior author (NCR) and a qualitative expert (MHM). Discussions focused on minimizing overlap between themes and guiding meaningful interpretation that remained grounded in the data. Representative quotes were selected, and themes were interpreted in light of the theoretical scaffold. Reflexive practices (e.g., journaling) and critical discussions with co-authors were utilized throughout the study to acknowledge the impactful role of researcher positionality and to heighten rigor.

### 2.4. Quality Criteria

Study rigor was enhanced by adhering to the four principles of quality in interpretive description [36]. Epistemological integrity (i.e., alignment of methods and assumptions with chosen epistemology) was considered by consulting with a qualitative expert (MHM) to ensure that methodological decisions, such as the use of purposive sampling and inductive data analysis approaches, aligned with constructivism. Representative credibility (i.e., that theoretical claims fit with how the data were obtained and analyzed) was addressed via purposive sampling to gather varied perspectives and prolonged engagement with participants throughout the intervention. Analytic logic (i.e., researcher logic is documented to ensure consistency between the research process and results) was attended to by maintaining a detailed record of analysis decisions and co-author discussions. Interpretive authority (i.e., ensuring researcher interpretations are trustworthy) was addressed by processing researcher thoughts and reactions in a reflexivity journal, as well as describing author positionality below.

### 2.5. Researcher Positionality

Given the active role of researchers in developing knowledge in interpretive description, the positionality of the first author, who conducted all interviews and had the primary role in the analysis, is acknowledged [36]. The first author is a 30-year-old white male who is able-bodied and has been physically active his whole life. Although the first author has no personal history of cancer, he has been a close witness to the impact of cancer. Furthermore, as an exercise oncology researcher, the first author is a firm believer in the value of PA for individuals living with and beyond cancer. He was personally invested in the study as part of his doctoral degree project, interacting directly with participants as the study coordinator during the 6-month PA intervention period prior to conducting interviews. The author’s prior interview experience includes qualitative research among advanced lung cancer populations and leading patient advisory board meetings within the EXCEL study [33,34,35].

## 3. Results

Of the 172 participants who completed the 24-week intervention period within the larger study, purposive sampling was used to invite 28 participants with varying demographic backgrounds, medical profiles, and physical activity histories to semi-structured interviews. Eighteen interviews were conducted, and 10 participants did not respond to the invitation. No reasons for refusal to participate were obtained. Participants were between 37 and 75 years old, with representation across seven Canadian provinces and territories; White, Indigenous, and South Asian identities; and eight cancer types (five with advanced cancer, nine on treatment) (Table 1). The average duration of self-monitoring app use was 18.7 ± 7.5 out of 24 weeks. Participants self-reported a median of 80.0, 210.0, and 225.0 moderate–vigorous PA minutes at baseline, 12 weeks, and 24 weeks, respectively. Fourteen participants completed the 24-week MVPA self-report, with eight decreasing and six increasing their MVPA during the PA maintenance period (i.e., weeks 12–24).

### 3.1. Themes

**Figure 1:**
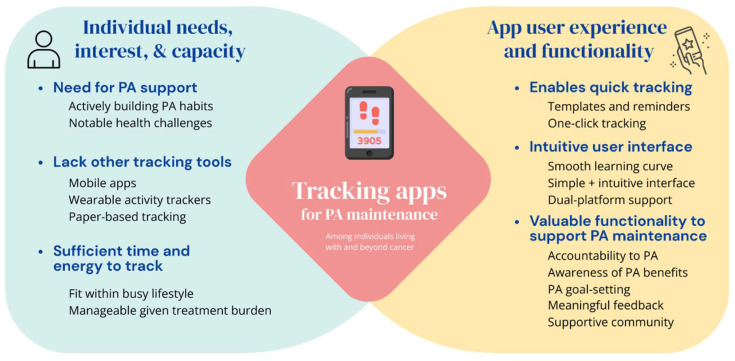
Considerations for using self-monitoring apps to support PA maintenance among individuals living with and beyond cancer.

Participants discussed their perspectives on the use and perceived value of the self-monitoring app to support PA maintenance. Figure 1 presents a visual summary of key takeaways from the present study. Four themes were developed, with illustrative quotes integrated into the results below.

#### 3.1.1. Theme One: Some Individuals Did Not Need the App to Stay Physically Active

Some participants explained that they did not need the self-monitoring app to support their PA habits either due to limited PA support needs or pre-existing PA tracking habits. For example, participants with many PA facilitators and few PA barriers were able to maintain PA levels without needing any additional PA support. “Part of the reason why I could let [the app] go like that is because I really felt that I didn’t need it in terms of my own self-discipline to continue on you know with exercise” (Participant 9, age 75, female). Specific facilitators noted by this group included well-established PA habits, high self-discipline for PA, as well as stable health status, thereby reducing the negative impact of the cancer-related PA barriers (e.g., cancer treatment, cancer- and treatment-related side effects) frequently reported among oncology populations.

I didn’t see any drastic changes so I kind of got bored with [it]. … I just got complacent in doing, entering it. Because I didn’t feel it was, my symptoms always were the same and my energy was always the same when I did it in the evening. … Somebody that’s in the journey as far as repairing or going through some of the different treatments, this would probably mean more.

(Participant 1, age 67, female.)

Continued use of the self-monitoring app was often limited among these individuals, given its lack of perceived usefulness for PA maintenance, which was achieved irrespective of app use. These participants did not need the study-specific self-monitoring app or other PA tracking tools to stay active.

Others indicated that they did not need the app due to the established use of tracking tools that worked well for them. While this group did express an interest in self-monitoring, their needs were met via paper- or app-based (e.g., Garmin Connect, Google Fit) PA and symptom tracking.

I like the idea that it’s paperless, but really I could just keep what I want to keep in my notes. Things that I want to track are so few, I mean sure it’s kind of nice to draw a graph. I do have a graph on graph paper of my weight. … And I keep coming back to the paper because it’s easy, it’s not time consuming. Even that takes me probably you know five minutes a day. Maybe 10.

(Participant 17, age 67, female.)

Therefore, the study-specific self-monitoring app was not perceived as valuable for these individuals, often leading to the abandonment of this app as they reverted to their preferred tracking tools. While participants’ discussions related to this theme were brief, given their limited PA support needs that resulted in limited app use, the findings highlight the importance of understanding needs on an individual level to determine whether a self-monitoring app may provide valuable PA maintenance support [31,32].

#### 3.1.2. Theme Two: Some Individuals Valued the App for Helping Them Maintain Their Physical Activity Habits

Several participants described how the self-monitoring app helped them to stay on track with their PA habits, especially during the PA maintenance period when there was a lack of scheduled exercise classes. Three prominent factors contributing to the perceived value of the app were discussed: increased accountability to perform regular PA, greater awareness of current PA and its health benefits, and prompts for PA goal setting.

Accountability to stay physically active

Regular self-monitoring in the app made some individuals more conscious of their current PA levels, motivating them to continue building and maintaining PA habits. One participant noted how the tracking itself became a habit, further reinforcing their PA habits.

It keeps me very conscious with the exercising and I think the Zamplo at nights when I’m having to record it—it’s just that more awareness. … Zamplo is just another way of that progression, that monitoring and working towards maintaining.

(Participant 1, age 67, female.)

I did it at seven o’clock so if I hadn’t done anything that was like maybe a bit of a prompt you put in zero minutes to be better than the next day, so I’d say you have some days I would’ve had no minutes, you feel a little bit guilty, so the next day you’d try a little bit harder.

(Participant 6, age 37, female.)

Daily notifications reminded some participants to first think about, then record their PA, energy, and fatigue. Furthermore, daily PA self-monitoring prompted self-reflection, often encouraging participants to plan for additional PA in the coming days. Accountability to PA stood out as the most frequently discussed sub-theme, emphasizing its importance to the perceived usefulness of a self-monitoring app for supporting PA maintenance in oncology.

Awareness of health benefits and the need to modify physical activity

A number of participants noted the value of recording and visualizing (i.e., via automated graphs) energy, fatigue, and other relevant health outcomes in the app. Having a visual overview of recent trends made them more aware of the positive benefits of PA, such as increased energy and decreased fatigue, which motivated them to stay physically active.

With Zamplo app showing me the energy level, I would go in not having a lot of energy and then sitting down to actually think about it and input it realizing that I had more energy and less fatigue [after exercising]. And then further on, I realized, okay, maybe I don’t have as much energy, but I’m going through a lot medically. … So for me, I look at Zamplo like a little lifeline and I just think that I have changed the way I think about exercise so much.

(Participant 18, age 63, female.)

Self-monitoring also prompted some participants to adapt their PA routines on days with worse symptoms (e.g., higher fatigue, poor sleep quality), allowing them to stay physically active despite their fluctuating health status.

Prompting physical activity goal-setting

A few participants also explained how the app’s weekly goal-setting prompts, combined with daily PA tracking routines, encouraged them to set PA goals and actively monitor them throughout the week. These personal PA goals (e.g., 5000 steps per day) served as an extrinsic motivator to stay physically active.

Okay, so I did that weekly [goal-setting] check-in usually on Sundays. … I was pretty consistent about doing it. And so, I was able to, at least see that over time, I was meeting that that step goal. … It was an easy goal to set in Zamplo and it was an easy goal to monitor in Zamplo, and because it was a single thing monitoring the graph is actually useful.

(Participant 16, age 58, female.)

As these participants reflected on weekly PA goals, meeting or exceeding them encouraged some individuals to maintain their PA and, as needed, increase their goals over time. Interestingly, only a small subset of participants mentioned that they used the PA goal-setting functionality, suggesting that many individuals used the app for regular self-monitoring but not for setting PA goals. Goal-setting appeared to be more relevant for individuals looking to build back up to their pre-diagnosis PA levels.

Adding value via extra feedback and support

While many participants emphasized the value of the app for supporting PA maintenance, others bemoaned the lack of meaningful feedback from the app, which negatively impacted continued use. A lack of automated data summaries or positive reinforcement via notifications, as well as challenges viewing graphs on mobile phones, contributed to these perceptions. As such, feedback must be both relevant and easy to understand to be perceived as a valuable self-monitoring app feature. Consistent with the technology acceptance model, the clear and understandable presentation of information can impact perceptions of both the ease of use and, indirectly, the usefulness of technology [31,32].

To address these concerns and increase the app’s potential value for supporting PA maintenance, participant requests included features such as automated summary reports of weekly tracking data and smart notifications, providing insights on PA and health trends.

Yeah like it just would have been nice to have like … a weekly summary or a monthly summary of whatever you were tracking. … I guess this would go more if it was like connected to a watch where you would get like a prompt like ‘this week you’ve only got this many minutes’ like almost like a motivating little quote or something right that would like come to your notifications.

(Participant 6, age 37, female.)

In addition, some participants spoke about the potential benefit of having a cancer exercise community to connect with directly in the app, providing valuable social support for PA during the PA maintenance phase. This suggestion was raised by participants living in remote locations who lacked social support and thus showed greater interest in engaging with an online community in the app.

I think for keeping people active, it would be nice to have also have maybe more of like a community. Where we could all be part of this community, and then we would use an app to not compete against each other, but to motivate each other. And I think that would be good because we’re all in the same physical challenges where we’re tired or fatigued, you know, that sort of thing. So, you know, you’d feel a little bit more on a level playing field with people in the same situation as yourself. … Especially for someone like me who’s rural and can’t do a lot like I don’t go anywhere with COVID because, you know, I have to be so careful.

(Participant 8, age 48, female.)

A common thread across these requests was the need for a self-monitoring app to provide meaningful output to participants via data-driven insights and encouragement, in return for the time and effort they spent entering data. Given prior experiences with “smart” PA apps featuring tailored feedback (e.g., Insights in Garmin Connect) or community support (e.g., Communities in Strava), many participants expected the study-specific self-monitoring app to do the same. These perspectives suggest that the provision of meaningful feedback can increase the perceived usefulness of PA self-monitoring apps, which may result in meaningful increases in long-term app usage [31,32].

#### 3.1.3. Theme Three: The User Experience Ranged from Intuitive to Confusing

Perspectives on the ease of use varied greatly between participants. Some individuals spoke about the importance of an intuitive user experience, especially to encourage prolonged app use throughout the study period. Past app experience made the self-monitoring app easier to learn, contributing to positive ease of use perceptions as individuals quickly became more skilled at using it. “So I think, for me, the reason it was kind of easy is because I’ve been using things like that already, right? I use apps that track various metrics, … I’m used to having to track things” (Participant 13, age 45, female). However, some individuals who considered themselves less “tech savvy” and had limited prior experience with apps also found the app intuitive to use. “When you initially look at it, it looked really busy, but it was fairly easy to navigate honestly and I am not technically savvy, I’ll tell you that” (Participant 10, age 47, female). Certain app features impacted these participants’ overall perceptions of the user interface. For example, study-specific tracking templates and dual-platform support (i.e., ability to use via both smartphone and computer) were key factors that enhanced usability, prompting continued app use.

In contrast, others had significant challenges with learning to use the self-monitoring app and navigating its basic functions. Many individuals in this group became overwhelmed due to the app’s user interface and high degree of customizability, noting that there were too many tracking options to choose from. “It’s almost like it was too open-ended for you to customize it, too customizable in a way that it was almost overwhelming. And for me personally, if I’m overwhelmed, I just put something down and don’t use it” (Participant 8, age 48, female). These challenges suggested that for individuals with both lower and higher technology literacy, the app was not always easy to learn, clear and understandable, or easy to use, three core elements of ease of use in the technology acceptance model [31,32]. Furthermore, they contributed to a time-intensive learning process, which was noted by many as a drawback to the app. When participants experienced difficulties learning to use the app or faced persistent problems over time, they grew increasingly frustrated and often abandoned the app.

To improve the ease of use, numerous participants recommended changes including a simplified user interface and the provision of additional guidance via in-app tutorials and pre-set PA tracking templates.

I wonder if it would be helpful to, like, have like [a little tutorial]—you start in one place—like explain to people ‘start here, start recording daily’. And then, after you get used to that, ‘okay now, if you want to start a weekly tracking or, whatever, and this is how you do that’.

(Participant 3, age 39, female.)

Tutorials may contribute to a smoother learning curve, helping participants better understand the app interface and functionality. Furthermore, the need for additional tracking templates was noted as a potential solution to counteract the sometimes overwhelming, open-ended nature of the present self-monitoring app.

Definitely having templates, … and it doesn’t have to be class-related, right? It can be anything, like your AM-PM check-in. ‘How are you doing?’ I think a lot of people would find that useful. You can track so much and for a brain like mine, it’ll shut itself down, because it’s like ‘well, I can track all of this stuff!’ And I think if you weren’t tech savvy that would be problematic too, right? Trying to figure out how to start [tracking].

(Participant 13, age 45, female.)

This theme highlighted the influence of the ease of use, or lack thereof, on continued use. Notably, these perspectives were often established within the early stages of using the app, indicating the importance of simplicity, in-app tutorials, templates, and sufficient technical support to address ease of use challenges.

#### 3.1.4. Theme Four: The Time Burden of App Use Ranged from Acceptable to Overwhelming

The time burden of using a self-monitoring app was relevant to many participants. Some participants appreciated that the self-monitoring app made tracking of PA and health outcomes quick and easy, allowing them to adhere to tracking habits despite having many other commitments.

For me really, truly it was like you know, during the dinner clean up, it was like a two-second put in your information, you know, get kids doing homework and it was super easy. So, I was like this is easy, and I can manage this.

(Participant 6, age 37, female.)

Reminder notifications and “one-click” tracking features (i.e., the “repeat from previous” button used to auto-fill information from the previous day of tracking) contributed to positive perceptions of the self-monitoring app and facilitated continued app use.

Other participants found the self-monitoring app too time-consuming to use, with the need for manual data entry due to a lack of automated tracking or synchronization with other technologies (e.g., with wearable activity trackers) discussed as notable downsides. Completing daily and weekly tracking presented a significant time burden, especially when participants had a broad range of PA and health factors to record.

So, I thought it would be pretty good but it was it was very time-consuming just to use it. It wasn’t simple. So that was the end. […] It took me a lot of time to figure out how to use it or I had to consult with somebody to use it and it just for the benefit, it wasn’t to me worth the time.

(Participant 17, age 67, female.)

This barrier was especially relevant for participants with busy schedules and lifestyle changes, such as going back to work or summer vacation, that disrupted their health tracking habits. Lifestyle barriers to PA self-monitoring were described in greater detail by younger participants balancing work and family responsibilities, as well as individuals currently undergoing cancer treatment, who had frequent appointments and greater health challenges (e.g., treatment side effects). Participants with greater time barriers struggled to fit the app into their daily routine.

Well, because I started work. I worked for the census, and I was just busy with that. … Plus, it was the summer and I went to the lake and our Internet is not good at the lake at all, and, so, that—that, you know, that made it... and, and I think when you’re in the holiday mindset you just take a break from stuff like that, so I did.

(Participant 5, age 61, female.)

The time burden negatively impacted the perceived ease of use for these participants, resulting in decreased intentions and actual use of the app. This is consistent with established social cognitive behavioral theories, wherein increased barriers are associated with diminished intentions and ultimately reduced behavior [32,45,46].

To reduce the time burden, many participants discussed the need for greater self-monitoring automation, as well as seamless integration with other PA-related technologies such as wearable activity trackers.

One of the frustrations that I had was that … I had to manually transfer information in from other places. And I realize to some extent that’s a security piece, … but I would have loved to be able to take the body battery function from [Garmin] and just cut and paste it over. Or not to cut and paste it over, just have it there.

(Participant 7, age 63, male.)

Ease of use improvements to simplify the user experience and reduce the time burden may, at least in part, address the challenges with app engagement during the PA maintenance period that were noted during the intervention.

## 4. Discussion

The current study highlighted the perspectives of remote and rural Canadians living with and beyond cancer on the potential of self-monitoring apps to support PA maintenance. It adds to the growing body of qualitative research on technology use for PA behavior change in oncology [23,24,25,26,27,28,29,30], filling knowledge gaps with respect to rural populations, prolonged engagement with technology, and the potential impact on PA maintenance. Whereas individuals with established PA habits and use of tracking tools saw less value in the app, others spoke about its value for keeping them accountable to PA, aware of the health benefits of PA, and prompting PA goal-setting. More personalized feedback and social support features were frequently suggested app improvements. The perceived ease of use, which varied widely from simple and intuitive to confusing and frustrating, acted as a concurrent facilitator and barrier to continued app use. Lastly, individuals emphasized the importance of quick and easy tracking in the app, with some individuals finding manual tracking too time consuming to fit within their busy lifestyles.

An emphasis on the ease of use as an influential factor for continued engagement with technology, as described in theme 3, was also identified as a key theme in prior qualitative evaluations after technology-based exercise oncology interventions [28,30]. These findings align with the technology acceptance model, wherein the perceived ease of use can impact actual use by changing an individual’s attitude towards technology [31,32]. As in the present project, Martin et al. reported that technical problems with the PA app and its time-consuming nature were key barriers highlighted during participant interviews [28]. Therefore, perceptions on the ease of use can either encourage or discourage further app use. While limited, these findings to date highlight that apps that are quick and easy to use may promote continued app engagement, which has been associated with improved PA maintenance in app-based interventions in adult populations [47,48]. The present study provides new insights on the potential temporality of factors impacting the continued use of technology, with perceived ease of use more frequently discussed in the context of early app adoption, whereas perceived usefulness was seen as more relevant to long-term use.

Regarding the perceived usefulness of technology for building and maintaining PA habits in oncology, common themes across qualitative studies include increased accountability to PA, as well as awareness of PA levels and the health benefits of PA [28,29,30]. For example, participants interviewed after a similar technology-based PA maintenance intervention that included health coaching, text messaging, and an activity tracker, mentioned how the intervention made them more accountable to staying active and continually reminded them of the positive benefits of PA [30]. However, as our results indicate, some individuals desire technology-based tools that not only track data, but also provide meaningful feedback and community support related to their health behaviors. Whereas technology is evidently a useful PA support tool for some, the present study emphasized that paper-based tracking methods are still preferred by others.

The present study shed light on factors impacting technology use among cancer populations that may limit their usefulness. Participants with limited time to use technology due to competing priorities, existing long-term PA habits and limited health concerns, and those using other tracking tools (i.e., other technology or paper-based tracking) saw limited value in using the self-monitoring app. A lower perceived value appeared to contribute to a lack of sustained app use over time. Better tailoring of technology-based support is thus needed to address individual needs and capabilities and improve engagement with technology, which may enhance the intervention’s effects on PA outcomes [15,48].

### 4.1. Implications for Future Research and App Development

Our study findings have several implications for future research and development work on technology to promote PA behavior change among cancer populations, with three key takeaways noted: (1) the need for tailored technology-based PA support, (2) the value of self-monitoring apps for PA behavior and suggestions for improving value, and (3) the impact of the ease of use and time burden on continued app use (see Figure 1).

First, given the varying PA support needs and perspectives on the value of technology to address them, a pre-intervention assessment may be useful to determine the type of PA support needed by an individual, informing better tailoring of PA maintenance support via technology. Further work to understand the interplay between factors impacting the perceived value (e.g., participant needs and preferences, PA barriers, and existing technology use) may be useful to inform this tailoring process.

Second, findings highlighted key factors (i.e., accountability to PA, awareness of PA benefits, PA goal-setting) contributing to the perceived usefulness of a self-monitoring app for supporting PA habits. An increased awareness of health benefits is especially relevant for individuals living with and beyond cancer as they seek to overcome treatment side effects and chronic symptoms such as cancer-related fatigue. In addition, more active support (e.g., summary reports, encouraging insights, in-app communities) was requested, which may make the app more useful for overcoming PA maintenance challenges. According to the technology acceptance model, perceived usefulness is the strongest predictor of actual technology use [31,32]. App developers and researchers are encouraged to consider these factors to optimize the impact of technology on PA maintenance in oncology.

Third, the ease of use is particularly important for apps aiming to support PA among cancer populations [25,28,30,49]. Whereas positive experiences encourage further use and allow individuals to discover an app’s value, significant early challenges often lead to app abandonment and preclude participants from realizing its full value. Based on the perspectives of the individuals living with and beyond cancer in the present sample, some of whom had limited experience with apps and lower technology literacy, it is advisable to simplify the user experience as much as possible to avoid frustration. Furthermore, given the busy lifestyles of these individuals, minimizing the time burden of app-based tracking (e.g., via automation and inter-app integration) is key to ensuring that self-monitoring remains feasible. Given the significant time burden of cancer treatment and follow-up care, as well as the common cancer-related cognitive challenges (e.g., chemo brain), ease of use considerations are likely to be even more relevant for individuals living with cancer than for healthy adults.

### 4.2. Strengths and Limitations

The key study strengths lie in our sampling approach and analyses. Purposive sampling was utilized to increase the demographic diversity of the sample in terms of age, gender, location, and cancer type. Sampling decisions also considered app use and PA levels over time, thus capturing understudied perspectives from individuals with low app engagement and those who did not maintain PA habits [50]. The analyses were enhanced by adhering to the quality criteria of interpretive description, with ongoing self-reflexive practices and multiple rounds of discussion with co-authors to remain cognizant of the author’s influence on the study findings and to avoid overinterpretation [36].

The study had several limitations. Despite the use of purposive sampling, the larger exercise oncology intervention from which we invited participants lacked variation in ethnicity and education level [33,34,35]. Most participants were female, white, and had moderate–high incomes and education levels. Furthermore, having the study coordinator conduct interviews may have adversely affected participant willingness to share negative perceptions during interviews. Lastly, the timing of the interviews, which were conducted 24 weeks after participants started using the self-monitoring app, made it difficult for participants to describe their early experiences with the app. Novel recruitment strategies and earlier interviews may help address these limitations in future work.

## 5. Conclusions

The present study sheds light on the potential value of a self-monitoring app to support PA maintenance among rural and remote Canadians living with and beyond cancer. The app was less suitable for participants with busy lifestyles, and not needed for those with established PA habits, limited health concerns to track, or a preference for other PA tracking tools. After a 24-week period of PA and health self-monitoring via the app, participants described how perceptions of ease of use impacted their decisions to continue using the app, with accumulating challenges often resulting in app abandonment. They discussed the value of the self-monitoring app for supporting PA maintenance by increasing accountability to PA, awareness of PA-related health benefits, and promoting PA goal- setting. Participants provided suggestions for improving the ease of use and perceived value of the self-monitoring app. These findings provide valuable insights to motivate additional research and inform the ongoing development of technology to help cancer populations stay physically active. Further research is needed to augment the present findings and address remaining challenges related to prolonged technology use and PA maintenance, especially among understudied cancer populations (e.g., rural, ethnic minorities, lower socioeconomic status, and advanced cancers).

## Figures and Tables

**Table 1:** Participant baseline characteristics, app use and usability ratings, and self-reported physical activity.

Total N = 18		
Age	N (%)	Mdn (Range)
30–39	2 (11.1)	59.5 (37–75)
40–49	4 (22.2)	
50–59	3 (16.7)	
60–69	8 (44.4)	
70–79	1 (5.6)	
Biological Sex	N (%)	
Female	14 (77.8)	
Male	4 (22.2)	
Location	N (%)	
Alberta	6 (33.3)	
British Columbia	2 (11.1)	
Saskatchewan	2 (11.1)	
Ontario	3 (16.7)	
Nova Scotia	2 (11.1)	
New Brunswick	2 (11.1)	
Northwest Territories	1 (5.6)	
Ethnic Identity	N (%)	
White	15 (83.3)	
South Asian	1 (5.6)	
Indigenous	2 (11.1)	
Marital Status	N (%)	
Married	10 (55.6)	
Divorced	3 (16.7)	
Separated	2 (11.1)	
Widowed	2 (11.1)	
Common Law	1 (5.6)	
Education	N (%)	
Some University	5 (27.8)	
Completed Undergraduate Degree	9 (50.0)	
Completed Graduate School	4 (22.2)	
Household Income	N (%)	
$20,000–$39,999	2 (11.1)	
$40,000–$59,999	3 (16.7)	
$60,000–$79,999	1 (5.6)	
$80,000–$99,999	5 (27.8)	
>$100,000	7 (38.9)	
Employment	N (%)	
Full-time	4 (22.2)	
Part-time	1 (5.6)	
Disability Leave	7 (38.9)	
Retired	6 (33.3)	
Cancer Type	N (%)	
Breast	8 (44.4)	
Lung	2 (11.1)	
Digestive	3 (16.7)	
Skin	2 (11.1)	
Gynecological	1 (5.6)	
Thyroid	1 (5.6)	
Genitourinary	1 (5.6)	
Blood	1 (5.6)	
Advanced	5 (27.8)	
Current Treatment	N (%)	
None	9 (50.0)	
Surgery	4 (22.2)	
Radiation	2 (11.1)	
Chemotherapy	1 (5.6)	
Immunotherapy	1 (5.6)	
Homeopathy	1 (5.6)	
Unknown	1 (5.6)	
Baseline Patient-Reported Outcomes	*M* (SD)	
Quality of Life (FACT-G, 0–108)	72.6 (19.9)	
Fatigue (FACIT-F, 0–52)	33.5 (12.5)	
Symptom Burden (ESAS, 0–100)	16.7 (12.6)	
Baseline Technology Use and Literacy	*M* (SD)	
Technology Use (0–10)	7.8 (2.1)	
eHealth Literacy (eHLQ, 1–4)	1.8 (0.2)	
App Usage and Ratings	*M* (SD)	
App Use, Weeks (0–24)	18.7 (7.5)	
App Use, Minutes	613.4 (607.0)	
App Ease of Use (MAUQ_E, 1–7)	4.8 (1.1)	
App Usefulness (MAUQ_U, 1–7)	4.6 (0.6)	
App Information Arrangement (MAUQ_I, 1–7)	4.9 (1.1)	
App Usability Total (MAUQ, 1–7)	4.8 (0.9)	
Self-Reported MVPA minutes (GLTEQ)	Mdn (IQR)	Range
Pre-diagnosis	125.0 (67.5–382.5)	0–780.0
Baseline	80.0 (5.0–247.5)	0–410.0
Week 12	210.0 (120.0–360.0)	0–870.0
Week 24	225.0 (125.0–290.0)	0–640.0
Week 12–24 change score	−70.0 (−120.0–120.0)	−230.0–200.0

FACT-G = Functional Assessment of Cancer Therapy—General [39]. FACIT-F = Functional Assessment of Chronic Illness Therapy—Fatigue [40]. ESAS = Edmonton Symptom Assessment Scale [41]. eHLQ = Electronic Health Literacy Questionnaire [42]. MAUQ = Mobile App Usability Questionnaire [43]. GLTEQ = Godin Leisure-Time Exercise Questionnaire [44]. IQR = interquartile range.

## Data Availability

The data presented in this study are available from the corresponding author upon reasonable request.
